# Addressing challenges of validity and internal consistency of mental health measures in a 27- year longitudinal cohort study – the Northern Swedish Cohort study

**DOI:** 10.1186/s12874-015-0099-6

**Published:** 2016-01-07

**Authors:** Anne Hammarström, Hugo Westerlund, Kaisa Kirves, Karina Nygren, Pekka Virtanen, Bruno Hägglöf

**Affiliations:** Department of Public Health and Clinical Medicine, Unit of Social Medicine, Umeå University, 901 85, Umeå, Sweden; Stress Research Institute, Stockholm University, Stockholm, Sweden; School of Social Sciences and Humanities, Psychology, University of Tampere, Tampere, Finland; Finnish Institute of Occupational Health, Helsinki, Finland; School of Health Sciences, University of Tampere, Tampere, Finland; Department of Clinical Sciences, Division of Child and Adolescent Psychiatry, Umeå University, Umeå, Sweden

**Keywords:** Mental health measures, Internal consistency, Validity, Longitudinal, Cohort study, Adolescence, Middle adulthood, Life course

## Abstract

**Background:**

There are inherent methodological challenges in the measurement of mental health problems in longitudinal research. There is constant development in definitions, taxonomies and demands concerning the properties of mental health measurements. The aim of this paper was to construct composite measures of mental health problems (according to today’s standard) from single questionnaire items devised in the early 1980s, and to evaluate their internal consistency and factorial invariance across the life course using the Northern Swedish Cohort.

**Methods:**

All pupils in the last year of compulsory school in Luleå in 1981 (*n* = 1083) form a prospective cohort study where the participants have been followed with questionnaires from the age of 16 (in 1981) until the age of 43 (in 2008). We created and tested the following composite measures from self-reports at each follow-up: depressive symptoms, anxiety symptoms, functional somatic symptoms, modified GHQ and positive health. Validity and internal consistency were tested by confirmatory factor analysis, including tests of factorial invariance over time.

**Results:**

As an overall assessment, the results showed that the composite measures (based on more than 30-year-old single item questions) are likely to have acceptable factorial invariance as well as internal consistency over time.

**Conclusions:**

Testing the properties of the mental health measures used in older studies according to the standards of today is of great importance in longitudinal research. Our study demonstrates that composite measures of mental health problems can be constructed from single items which are more than 30 years old and that these measures seem to have the same factorial structure and internal consistency across a significant part of the life course. Thus, it can be possible to overcome some specific inherent methodological challenges in using historical data in longitudinal research.

## Background

There are inherent methodological challenges in the measurement of mental health problems in longitudinal research. Mental health problems represent a variety of symptoms or diagnoses with a wide range of severity, from minor reversible reactions to lifelong severe disorders. There is constant development in definitions of mental health problems and in what is demanded of the properties of mental health measures. Also, mental health problems are defined and described in different terms and taxonomies by period and age. In addition, adolescents may understand and express the same mental health problems in a different way from adults [1, 2]. To enable comparisons over time, longitudinal studies need to keep the initial questions, while additional age-relevant and up-to-date measures may be difficult to include due to the need to keep the questionnaires short. For example, at the beginning of the 1980s there was a lack of validated measures of mental health problems among adolescents. The standard of that time was to interview teachers or parents (up to the age of 18) [3, 4]. A Norwegian child psychiatrist was one of the few known researchers in Scandinavia at that time who directed questionnaires to young people themselves about their mental health [5]. At the time, mental health was measured with single item questions. The standard of today in self-reports on mental health is composite measures of the presence of mental health problems, for example DSM and ICD related symptom clusters or broader symptom clusters, e.g., emotional problems, conduct problems or even broader dimensions reflecting internalised or externalised symptoms [6–8]. Thus, DSM oriented questionnaires have been developed during recent years with dimensions of affective, anxiety and conduct problems [8, 9] as well as broader dimensions of symptom domains such as internalised and externalised problems. Internalised problems represent depressive symptoms, anxiety, and functional somatic symptoms (FSS), whereas externalised problems describe different symptoms of out-acting behaviour such as antisocial, delinquent and aggressive behaviour [8, 10]. There is also a positive dimension of mental health which is more than the absence of mental health problems [11]. A question that remains to be analysed is whether measures of more modern constructs of mental health symptoms can be derived from old single items as well as whether the psychometric properties of such measures are acceptable across the life course.

The aim of this paper was to construct composite measures of mental health problems from single item questions about such problems from the early 1980s which conform to contemporary measurement standards with items largely parallel to the criteria in the DSM diagnostic system [12] and constructs from internationally validated self-report questionnaires [8, 13]. The aim was further to evaluate the internal consistency and factorial invariance of these composite measures from adolescence to middle age using the Northern Swedish Cohort.

## Methods

### Population

The population consists of all pupils in the last year of compulsory school (ninth grade) in the municipality of Luleå in Northern Sweden in 1981 [14]. The attrition rate has been extremely low. Of the total 1083 pupils (506 girls, 577 boys) who were invited, 1080 participated in the baseline investigation. Of those still alive at the latest follow-up in 2008 (*n* = 1071) 1010 still participated, meaning a response rate of 94.3 %. In the final analyses of this paper, the sample size varied between 914 and 934 individuals due to missing values. The missing data were handled with maximum likelihood estimation provided by Mplus. Of the 934 participants in 2008, 44.1 % were women and 34.9 % were blue-collar workers, 13.6 % lower white-collar, and 51.6 % upper white-collar workers. Moreover, 57.9 % rated their general health as good, 4.5 % as bad, and 28.1 % evaluated themselves as having in between good and bad health.

### The cohort

The initial aim of the Northern Swedish Cohort was to analyse the health consequences of youth unemployment. Thus, the questionnaires from the start have contained a large number of questions about both somatic and mental health symptoms. The cohort has been shown to be representative of Sweden as a whole in relation to demographics, socio-economic status and health complaints [14] and also representative of Scandinavian young people in relation to self-reported mental health symptoms [15].

### Data collection

The cohort has been investigated with extensive questionnaires from the start at age 16 (T1), with follow-ups at ages 18, 21 (T2), 30 (T3), and 43 years (T4). The questionnaires were collected during school hours at age 16 and at school class reunions at the follow-ups. The questionnaire was mailed to those who could not participate in these reunions. A shorter questionnaire was also conducted at age 18 (T1 for the General Health Questionnaire (GHQ) variables as described below). During all investigations, the participants completed questionnaires that included questions about different mental and somatic symptoms, health behaviours, socio-economic status, employment etc.

Mental health problems and somatic symptoms were measured with the same questions during the whole follow-up. The only exception was GHQ which was first included at age 18.

Ethical approval was obtained for the whole follow-up by Uppsala and Umeå University, as well as by the Regional Ethical Review Board in Umeå. Written consent has not been requested from these committees. The respondent is regarded as giving written consent when answering the questionnaire. Participants were able to opt out at any time simply by not completing any of the waves of the survey.

### Measures

We use the term questionnaire item to denote an individual question that the respondents have answered in the questionnaire by a single response. By measure (or composite measure) we mean a set of items that are thought to represent the same latent concept (e.g., depressive symptoms). A factor denotes a statistical variable which summarises variance shared between a number of observed variables, e.g., responses to questionnaire items, potentially corresponding to an underlying, unobserved latent variable. The extent to which the observed variance of the individual items in a theoretically constructed (composite) measure can be described by such a factor is an indication of the internal consistency of the measure.

When the study started at the beginning of the 1980s we found no validated measures of mental health directed towards young people themselves. Instead, we were inspired by the single item questions about mental health symptoms used by a Norwegian child psychiatrist in his studies of 16-year-old pupils [5]. All items (including response distribution at T1, response options and their coding) of the measures are described in detail in Table 1.

**Table 1:** Description of all questions included in the mental health measures

Questionnaire items	Response options (coding; distribution at T1, i.e., age 16)	Inclusion in
		AS = Anxiety Symptoms
		DS = Depressive Symptoms
		FSS = Functional Somatic Symptoms
Have you have experienced any nervous problems in the past 12 months?		
Restlessness	*Ticked box* (1*; 15.9 %) *Unticked box* (0*; 84.1 %)	AS
Concentration difficulties	*Ticked box* (1*; 15.9 %) *Unticked box* (0*; 84.1 %)	AS, DS
Worried or anxious	*Ticked box* (1*; 12.1 %) *Unticked box* (0*; 87.9 %)	AS
Palpitations or stomach problems	*Ticked box* (1*; 6.6 %) *Unticked box* (0*; 93.4 %)	AS, FSS
Anxiety or panic	*Ticked box* (1*; 4.0 %) *Unticked box* (0*; 96.0 %)	AS
How often have you experienced any nervous problems during the past 12 months?	*Never* (*1; 72.7 %), *Off and on* (*1; 25.9 %), *Quite often* (*2; 1.4 %), *All the time* (*2; 0 %)	
Does it happen that you feel dejected when you think about the future?	*Almost never* (0; 31.5 %), *No, more seldom* (1; 45.7 %), *Yes, quite often* (1; 20.3 %), *Yes, very often* (2; 2.5 %)	DS
How often have you felt sad or depressed during the last 12 months?	*Never* (0; 27.4 %), *Off and on* (1; 66.0 %), *Quite often* (1; 6.2 %), *All the time* (2; 0.4 %)	DS
Have you experienced sleeping problems during the last 12 months?	*Never* (0; 63.1 %), *Off and on* (1; 33.4 %), *Quite often* (1; 2.7 %), *All the time* (2; 0.9 %)	DS, FSS
Have you now (or have you during the past 12 months had) any of the following diseases or symptoms?		
Poor appetite	*No* (0; 75.8 %), *Yes, moderate* (1; 22.2 %), *Yes, severe* (2; 2.0 %)	DS
Headache, migraine	*No* (0; 47.0 %), *Yes, moderate* (1; 48.1 %), *Yes, severe* (2; 5.0 %)	FSS
Other stomach ache (than heartburn, gastritis or gastric ulcer)	*No* (0; 66.3 %), *Yes, moderate* (1; 32.3 %), *Yes, severe* (2; 1.4 %)	FSS
Nausea	*No* (0; 53.8 %), *Yes, moderate* (1; 44.6 %), *Yes, severe* (2; 1.6 %)	FSS
Backache, hip ache or sciatica	*No* (0; 74.0 %), *Yes, moderate* (1; 23.5 %), *Yes, severe* (2; 2.5 %)	FSS
Tiredness/fatigue	*No* (0; 43.8 %), *Yes, moderate* (1; 50.2 %), *Yes, severe* (2; 6.0 %)	DS, FSS
Breathlessness	*No* (0; 81.9 %), *Yes, moderate* (1; 16.6 %), *Yes, severe* (2; 1.5 %)	FSS
Dizziness	*No* (0; 80.7 %), *Yes, moderate* (1; 18.2 %), *Yes, severe* (2; 1.1 %)	FSS
Overstrain	*No* (0; 84.8 %), *Yes, moderate* (1; 14.3 %), *Yes, severe* (2; 0.9 %)	FSS

*Denotes multiplication between the response to an item about presence of a symptom and a later about the frequency of the set of symptoms

Inspired by the diagnostic symptom criteria of depression and anxiety disorders of the DSM system [12], syndrome and DSM oriented domains of the YSR (Youth Self-report) scale [8] and subscales from SDQ (Strengths and Difficulties Questionnaire) self-report scale [6], we recently developed measures of anxiety symptoms, depressive symptoms and FSS. In accordance with YSR we also combined measures in broader domains of internalising symptoms [8].

#### Anxiety symptoms

The measure of anxiety symptoms included the following five symptoms: restlessness; concentration difficulties; worry or anxiety; palpitations or stomach problems; and anxiety or panic. Respondents who had checked “No” for all symptoms received a total measure value of 0, which is also the value assigned to each unchecked symptom. A follow-up question asked about frequency. For respondents who had indicated a frequency of “Off and on” or “Never” together with one or more of the individual symptoms, each checked symptom was recoded to 1, whereas for those who had indicated “Often” or “All the time” each checked symptom was recoded to 2. The measure value was finally computed as the mean of the five recoded item values with a theoretical range of 0–2. For example, someone who had first indicated that they had experienced restlessness and palpitation and then answered that (s)he had had such symptoms often, received the total score of (1*2 + 0*2 + 0*2 + 1*2 + 0*2)/5 = 0.8 for anxiety symptoms.

#### Depressive symptoms

Depressive symptoms were measured with six symptoms: sleeping problems (0–3), poor appetite (0–2), general tiredness (0–2), feeling down and sad (0–3), dejected about the future (0–3) and concentration difficulties (0–2 after recoding as explained under Anxiety symptoms above). Response options ranging from 0 to 3 were recoded to 0–2 by combining the two middle response options. The measure value was finally computed as the mean of the six recoded item values.

FSS was constructed by a panel consisting of 25 experienced clinical psychologists, paediatricians and child and adult psychiatrists. For each of 42 listed symptoms, the panel was asked to judge whether they considered it to belong to FSS or not. The following ten symptoms received the highest number of yes answers: headache or migraine (80 % agreed); other stomach ache (than heartburn, gastritis or gastric ulcer; 96 % agreed); nausea (68 %); backache, hip pain or sciatica (64 %); general tiredness (76 %); breathlessness (64 %); dizziness (72 %); overstrain (64 %); sleeping problems (68 %); and palpitations (72 %). “Tiredness” and “sleeplessness” are the same items, coded in the same way as in measure of depressive symptoms. “Palpitations” is the same item, coded in the same way as in the measure of anxiety symptoms. All other items were coded as 0–2. The measure value was finally computed as the mean of the ten recoded item values.

A modified version of GHQ12, *Negative GHQ* (GHQ6-n), was constructed from the following six items from the GHQ12 measure [16]: sleeping problems, feeling tense and strained, feeling unhappy and depressed, finding it hard to deal with problems, feelings of lost confidence, and finally feeling worthless. All items were coded as 1 (not at all), 2 (usual), 3 (somewhat more than usual), and 4 (much more than usual). The measure value was computed as the mean of the six recoded item values.

GHQ was translated into Swedish in the early 1980s by the cohort researchers, who tried to adapt the scale to young people by modifying the response options for six of the questions. From these, a *Positive GHQ* (GHQ6-p) was created based on: ability to concentrate, feeling useful, making decisions, enjoying daily activities, solving problems and being reasonably happy. All items were coded as the modified GHQ6-n measure above. The measure value was computed as the mean of the six recoded item values.

For the latter two scales T1 refers to age 18 as GHQ was not included at the baseline investigation.

### Data analysis

First, the factor structure of each measure (i.e., anxiety symptoms, depressive symptoms, FSS, and the two GHQ measures) was tested separately in each year with measurement models. A measurement model is a model that examines the relationship between the latent factors and items related to them. Confirmatory factor analyses (CFA) were conducted with robust weighted least squares estimator (WLSMV) because all the items were categorical [17]. The fit of the measurement models was evaluated using χ^2^, the Comparative Fit Index (CFI), the Root Mean Square Error of Approximation (RMSEA) and its 90 % confidence interval, and factor loadings. A good fit was indicated by a non-significant χ^2^, CFI ≥ .95, RMSEA ≤ .06 and loadings ≥ .40 as suggested by Hu and Bentler [18]. However, the χ^2^ is sensitive to sample size, meaning that it nearly always rejects the model when large samples are used [19].

Second, the factorial invariance (over time) of the measures was tested [20] by comparing two models separately for each measure in a freely estimated and a constrained longitudinal measurement model. Factorial invariance was tested at the level of factor loadings in order to verify that the same manifest items were measuring the same latent attributes (e.g., anxiety symptoms) in the same way in each year. In the freely estimated model, all factor loadings were freely estimated, while in the constrained model equality constraints were imposed on the corresponding factor loadings across the four measurement times. In both models, the corresponding measurement errors of the original items were allowed to covary across time. The difference in goodness-of-fit of the freely estimated and the constrained model was compared with the χ^2^-difference test. The factorial invariance was supported if the χ^2^-difference test produced a non-significant loss of fit when the factor loadings were constrained to be equal across time.

Third, the possibility to form a measure of internalised mental health problems (including anxiety and depressive symptoms) and a measure of extended internalised mental health problems (including anxiety symptoms, depressive symptoms and FSS) was investigated by comparing the following three models separately at each time point: A) a three-factor model (i.e., anxiety symptoms, depressive symptoms and FSS), B) a two-factor model (i.e., internalised mental health problems and FSS), C) a one-factor model (i.e., extended internalised mental health problems). The alternative nested models (i.e., A vs. B and B vs. C) were compared by fit indices and the χ^2^-difference tests. The significantly lowest χ^2^ was chosen.

The analyses were performed using the Mplus statistical package (Version 7.3).

The internal consistency of the scales was investigated with Cronbach’s alpha (α) using IBM SPSS Statistics program (Version 21). A good internal consistency was indicated by .70 ≤ α < .90 and an acceptable internal consistency by .60 ≤ α < .70 [21].

## Results

### Factor structure of the mental health measures

Table 2 shows the fit indices of the measurement models and Cronbach’s alpha for each measure at each time point. In terms of the CFI, the fit of all models was good. However, the RMSEA was above .06 in the models of anxiety symptoms at T2 and at T4 (.10) and GHQ at T1–T4 (.07–.14). Nevertheless, when the RMSEA is between .08 and .10, it indicates that the fit is still acceptable [22]. Consequently, based on these results, the factor structure of anxiety symptoms, depressive symptoms and FSS can be seen as acceptable at each time point.

**Table 2:** The fit indices of the measurement models and Cronbach’s alpha for each measure (anxiety symptoms, depressive symptoms, FSS, and GHQ) at each time point

Scale	Wave	*N*	*χ* ^2^	Df	*P*	CFI	RMSEA	90 % CI for RMSEA	Range of factor loadings	Cronbach’s alpha
Anxiety symptoms	T1	928	18.45	5	.002	.99	.05	.03–.08	.62–.85	.72
	T2	929	48.20	5	<0.001	.98	.10	.07–.12	.80–.91	.79
	T3	914	22.42	5	<0.001	1.00	.06	.04–.09	.86–.98	.90
	T4	929	51.46	5	<0.001	.99	.10	.08–.13	.82–.92	.86
Depressive symptoms	T1	929	18.45	9	.030	.99	.03	.01–.06	.44–.80	.65
	T2	929	5.78	9	.762	1.00	.00	.00–.03	.44–.74	.61
	T3	921	19.66	9	.020	.99	.04	.01–.06	.55–.79	.67
	T4	929	34.07	9	<0.001	.99	.06	.04–.08	.62–.89	.76
FSS	T1	929	85.29	35	<0.001	.97	.04	.03–.05	.43–.67	.70
	T2	929	111.67	35	<0.001	.95	.05	.04–.06	.47–.68	.70
	T3	921	101.52	35	<0.001	.97	.05	.04–.06	.47–.75	.74
	T4	929	154.35	35	<0.001	.96	.06	.05–.07	.48–.79	.79
GHQ6-n	T1	926	165.42	9	<0.001	.97	.14	.12–.16	.64–.89	.81
	T2	927	169.88	9	<0.001	.98	.14	.12–.16	.66–.95	.83
	T3	916	180.78	9	<0.001	.99	.14	.13–.16	.62–.97	.84
	T4	923	235.61	9	<0.001	.98	.17	.15–.18	.68–.96	.85
GHQ6-p	T1	927	53.21	9	.000	.98	.07	.06–.09	.45–.76	.72
	T2	927	56.44	9	.000	.98	.08	.06–.10	.45–.79	.72
	T3	917	92.58	9	.000	.98	.10	.08–.12	.61–.85	.79
	T4	926	155.40	9	.000	.96	.13	.12–.15	.58–.85	.80

With regard to GHQ (both GHQ6-n and GHQ6-p) the situation is more complicated. Although many of the RMSEA values were above .10, almost all the factor loadings were statistically significant and above .40 at each time point. Moreover, the models did not produce any large modification indices, which would have indicated possible ways to modify the model in order to reduce RMSEA. In addition, as shown in Table 3, the longitudinal measurement models of GHQ had the RMSEA values of .05 (GHQ6-n) and .03 (GHQ-p). This indicates that the possible problems with the factor structures of GHQ6-n and GHQ-p disappeared when it was modelled over time (T1–T4). Thus, the two measures of GHQ also seem to have an adequate factor structure.

**Table 3:** The freely estimated (A) and the constrained (B) longitudinal measurement models for each measure (*N* = 934)

Scale	Model	*χ* ^2^	Df	*p*	CFI	RMSEA	90 % CI for RMSEA	Range of factor loadings	Comparison	∆*χ* ^2^	∆df	*P*
Anxiety symptoms T1–T4	A	262.42	136	<0.001	.99	.03	.03–.04	.68–.96				
	B	270.60	148	<0.001	.99	.03	.02–.04	.73–.95	A vs. B	21.41	12	.045
Depressive symptoms T1–T4	A	264.95	210	.006	.99	.02	.01–.02	.41–.91				
	B	290.00	225	.002	.99	.02	.01–.02	.50–.90	A vs. B	24.94	15	.051
FSS T1–T4	A	974.82	674	<0.001	.97	.02	.02–.03	.42–.78				
	B	978.29	701	<0.001	.97	.02	.02–.02	.46–.77	A vs. B	38.24	27	.074
GHQ6-n T1–T4	A	783.86	212	<0.001	.98	.05	.05–.06	.61–.97				
	B	753.92	227	<0.001	.98	.05	.05–.05	.62–.97	A vs. B	24.43	15	.058
GHQ6-p T1–T4	A	429.71	210	<0.001	.98	.03	.03–.04	.44–.84				
	B	480.69	225	<0.001	.97	.04	.03–.04	.53–.84	A vs. B	55.54	15	.000

In line with the results of CFA, the internal consistency (Cronbach’s alpha) of all measures at each time point ranged from acceptable to good.

### Factorial invariance over time

Table 3 presents the freely estimated and the constrained longitudinal measurement models for each measure. All freely estimated longitudinal models (models A) had a good fit. Although the excellent values of the fit indices (CFI and RMSEA) of anxiety symptoms did not change after the constraints were added, the statistically significant χ^2^-difference test still indicated that the fit of the model decreased (*p* = .045). Because of the excellent fit, the statistically significant χ^2^-difference test may be ignored and the factor structure of the measure of anxiety symptoms can be seen as invariant over time. With regard to depressive symptoms, FSS, and GHQ6-n, both the fit indices and the χ^2^-difference tests indicated that the factor structures of these measures were invariant over time. However, a small decrease in the model fit and a statistically significant χ^2^-difference test indicated that the factor structure of GHQ6-p was not invariant across time.

### Combined measures

First, in order to test whether a measure of internalised mental health problems (including anxiety symptoms and depressive symptoms) or a measure of extended internalised mental health problems (including anxiety symptoms, depressive symptoms, and FSS) could be formed, each of the four items that were shared by these three measures needed to be classified into one of the measures. Based on the fit of the different models and modification indices (not reported here), the final best-fitting and theoretically grounded three-factor model was the following. Anxiety symptoms consisted of the original five items (i.e., restlessness, concentration difficulties, worry or anxiety, palpitations, and anxiety or panic). Depressive symptoms consisted of the five items which remained when “concentration difficulties” was included in anxiety symptoms (i.e., sleeplessness, poor appetite, tiredness, feeling down and sad, and dejected about the future). FSS consisted of the seven items which remained when “palpitations” was included in anxiety symptoms, and “tiredness” and “sleeplessness” were included in depressive symptoms (i.e., headache or migraine, other stomach ache, nausea, backache or hip pain, breathlessness, dizziness, and overstrain).

Next, three different factor models (i.e., A = anxiety symptoms, depressive symptoms, and FSS; B = internalised mental health problems and FSS; C = extended internalised mental health problems) were analysed and compared between each year. As Table 4 shows, the three-factor model (A) was the best model at each point of time in terms of the fit indices and the χ^2^-difference tests. Thus, it seemed as if the measures of anxiety symptoms, depressive symptoms, and FSS were separate constructs that should not be integrated. Nevertheless, the fit of the three-factor and the two-factor model at T4 was the same in terms of the CFI and the RMSEA, which implies that the measure of internalised mental health could be formed at T4 without a significant loss of fit.

**Table 4:** Comparison of factor models (A = three-factor, B = two-factor, C = one-factor) at T1–T4

Model		*N*	*χ* ^2^	df	*p*	CFI	RMSEA	90 % CI for RMSEA	Range of factor loadings	Comparison	∆*χ* ^2^	∆df	*p*
T1	A	929	242.13	116	<0.001	.97	.03	.03–.04	.39–.85				
	B	929	316.70	118	<0.001	.95	.04	.04–.05	.39–.82	A vs. B	48.36	2	<0.001
	C	929	427.24	119	<0.001	.92	.05	.05–.06	.33–.81	B vs. C	62.14	1	<0.001
T2	A	929	239.75	116	<0.001	.98	.03	.03–.04	.42–.90				
	B	929	363.63	118	<0.001	.95	.05	.04–.05	.41–.87	A vs. B	53.43	2	<0.001
	C	929	491.39	119	<0.001	.93	.06	.05–.06	.42–.86	B vs. C	59.28	1	<0.001
T3	A	921	335.01	116	<0.001	.99	.05	.04–.05	.45–.97				
	B	921	491.34	118	<0.001	.98	.06	.05–.06	.45–.98	A vs. B	71.88	2	<0.001
	C	921	635.43	119	<0.001	.98	.07	.06–.07	.39–.98	B vs. C	59.04	1	<0.001
T4	A	929	439.58	116	<0.001	.97	.06	.05–.06	.48–.92				
	B	929	485.53	118	<0.001	.97	.06	.05–.06	.48–.91	A vs. B	29.99	2	<0.001
	C	929	666.37	119	<0.001	.95	.07	.07–.08	.42–.90	B vs. C	84.05	1	<0.001

*Note*: A = the factors of anxiety symptoms, depressive symptoms, and FSS; B = the factors of internalised mental health and FSS; C = the factor of extended internalised mental health

## Discussion

The study showed that it was possible to form composite measures of mental health problems from single item questions regarding anxiety symptoms, depressive symptoms and FSS with acceptable to good internal consistency and factorial invariance across the different follow-ups. For the modified GHQ measures, the psychometric properties were less good but still acceptable at the different follow-ups.

Another possibility of describing symptoms is the dimensional approach, i.e., by combining a broader spectrum of symptoms. Internalised mental health problems can include both depressive and anxiety symptoms as well as FSS in line with some questionnaires [7, 13]. In our analyses we found that keeping anxiety symptoms, depressive symptoms and FSS in separate domains showed better psychometric properties than a combination of two or three of them.

GHQ differs from the rest of the studied composite measures in that it is based on an established measure [16]. Also its validity in detecting “cases” of “non-psychotic psychiatric disease” has already been established [23]. Our analysis showed that there were problems in the factor structure of GHQ when used as a simple score, but they disappear when modelled over time. In other words, in a cross-sectional setting it is preferable to use GHQ as a dichotomous screening instrument while in longitudinal settings it seems to be possible to use it as a scale.

There are problems in longitudinal cohort studies as informants grow older and develop, as culture and society differ through time and as the same items might have different meanings over time. In spite of that we found rather good factor structure invariance across time, indicating that the four measures do capture the same underlying phenomena at all the studied ages from 16 to 43 years.

Placing our findings in a wider context, our analysis provides an innovative approach and could be an inspiration for both old and newer cohorts. Many of the other old public health oriented cohort studies from the early 1980s included, at least in their first wave(s), single items about mental health symptoms, rather than clinical investigations or validated measures. This is the case for the Isle of Wight study [24], the 1958 British birth cohort [25], the Nord-Trøndelag Health Study (known as the HUNT Study) [26], the Tampere cohort study of school leavers [27], and the US Wisconsin Longitudinal Study [28]. However, the consistency between data collections is far lower for several of these cohorts, which means that longitudinal analyses of composite measures of mental health would be more difficult to perform. As in our study, the National Longitudinal Study of Youths from the US [29, 30] identified that the factor structure of anxiety and depressive symptoms was invariant over time in a population of children between 4 and 14 years of age. Overall, we argue that our work could be useful for several of the existing old cohort studies. Also, our paper is an inspiration for newer cohorts to keep their initial questions over time.

### Strengths and limitations

One of the major strengths of the Northern Swedish Cohort study has been its extraordinarily high response rate. In the last follow-up, 94.3 % of those still alive participated in the study. As a result, the cohort includes a group of people who otherwise are hard to reach [31], e.g., due to poor health, where mental health problems interfere with their ability or willingness to respond to questionnaires, threatening the representativeness of the findings. Moreover, although the data come mainly from one region in Sweden, the cohort has been shown to be representative of the country as a whole in critical respects [14].

A possible limitation is that, although CFA was developed to study the structure of a proposed measure, it is often criticised because of the fit indices and their vague cut-off values [32]. However, these problems are most pronounced in small datasets, and since our data consist of more than 900 respondents, we see CFA as the most appropriate method to investigate the structure of the proposed mental health measures.

Analysing the responsiveness, the extent to which the composite measures are able to detect changes over time in the phenomena that the measures are intended to reflect is, unfortunately, not possible in the data that we have access to, since it would require some kind of external criterion of the real change (for instance repeated psychiatric examinations). However, we would argue that the correspondence between the items in the composite measures with current concepts of mental problems makes it reasonably plausible that changes would be detected.

Although the content validity of our mental health measures cannot be analysed empirically it can be assessed in relation to the categorical diagnostic criteria of DSM 5 [33]. All six symptoms of our measure of depressive symptoms are within the nine DSM 5 criteria for major depression and therefore we consider the content validity to be high. The following four symptoms from DSM 5 were not available in our questionnaires: diminished interest or pleasure; psychomotor agitation or retardation; feelings of worthlessness or guilt; thoughts of death, suicidal ideation or attempts, or suicidal plan. The depressive symptoms in our measure capture common depressive symptoms while e.g., psychomotor agitation or retardation and thoughts of death, suicidal ideation or attempts, or suicidal plan represent symptoms associated with more severe depression [34]. Our measure of depressive symptoms is not aimed at diagnosis of depression, but since six of our depressive items are represented in the DSM 5 diagnostic criteria and are common symptoms of major depression we consider our measure of depressive symptoms to have good content validity.

Our measure of anxiety symptoms represents rather broad aspects of anxiety. “Worried or Anxious” and “Anxiety or Panic”, which are included in our measure, are main criteria for most anxiety syndromes of DSM 5. “Restlessness” and “Concentration difficulties” are symptoms in General Anxiety Disorder. “Palpitation or stomach problems” are symptoms of both social anxiety disorder and panic disorder. The face validity of our measure is high since similar items are included in the validated measure of anxiety in the Hospital, Anxiety and Depression Scale [35].

FSS is a complex concept and there is an ongoing debate about its nature, diagnosis and impact [36]. As described above, we used a panel in order to construct our FSS measure and thus the face validity of our measure is high. The symptoms of our measure also correspond well with what most researchers agree upon [37–39]. Support for the predictive validity of our measure was found in a study of FSS among 16-year-old pupils which showed that FSS can predict severe adult mental health disorders [40]. DSM 5 cannot be used as comparison as its main focus of the corresponding diagnosis (Somatic Symptom Disorder) is on all possible somatic symptoms which are distressing or disruptive of daily life.

In summary, the same or similar items can be found in different self-reported measures that assess depressive symptoms, anxiety and FSS symptoms as well as in categorical diagnostic systems such as DSM. Also, symptom criteria for depressive symptoms and anxiety disorders are almost identical according to the DSM manual from mid adolescence up to adulthood. Therefore, we believe that the content validity of our measurements on depressive symptoms, anxiety symptoms and FSS is good.

We would furthermore argue that the content validity of the measures of depressive and anxiety symptoms as well as of FSS is acceptable due to face validity and a relatively close correspondence between the included items and internationally used self-report scales and the DSM 5 criteria for depression and anxiety. As regards functional somatic symptoms, the symptoms included in our FSS scale are commonly found in measurements of FSS in children and adults. There is, however, no clear gold standard for FSS.

A limitation of the paper is the lack of a quantitative assessment of criterion validity. This will, however, be analysed in an ongoing study where the measures presented in this paper are validated in a clinical population of youths who are diagnosed according to the DSM 5 system combined with self-reports on mental health problems by young people (YSR, SDQ).

## Conclusions

Testing the properties of the mental health measures used in older studies according to the standards of today is of great importance in longitudinal research. The main implication of our study is that composite measures of mental health problems can be constructed from single items which are more than 30 years old and that these measures seem to have the same factorial structure and internal consistency across a significant part of the life course. Thus, it can be possible to overcome some specific inherent methodological challenges in using historical data in longitudinal research.

Our recommendations for old cohorts are to stick to their original questions about mental health symptoms and to test their validity as composite measures.
